# Telomere Length as a Biomarker for Adiposity Changes after a Multidisciplinary Intervention in Overweight/Obese Adolescents: The EVASYON Study

**DOI:** 10.1371/journal.pone.0089828

**Published:** 2014-02-24

**Authors:** Sonia García-Calzón, Adriana Moleres, Ascensión Marcos, Cristina Campoy, Luis A. Moreno, M. Cristina Azcona-Sanjulián, Miguel A. Martínez-González, J. Alfredo Martínez, Guillermo Zalba, Amelia Marti, on behalf of the EVASYON Study Group

**Affiliations:** 1 Department of Nutrition, Food Science and Physiology, University of Navarra, Pamplona, Spain; 2 Immunonutrition Research Group, Department of Metabolism and Nutrition, Institute of Food Science, Technology, and Nutrition, Instituto del Frío, Spanish National Research Council, Madrid, Spain; 3 Pediatric Department, Medicine School, Universidad de Granada, Granada, Spain; 4 GENUD (Growth, Exercise, Nutrition and Development) Research Group, Facultad de Ciencias de la Salud, University of Zaragoza, Zaragoza, Spain; 5 Pediatric Endocrinology Unit, Department of Pediatrics, University of Navarra Hospital, Pamplona, Spain; 6 Department of Preventive Medicine and Public Health, University of Navarra, Pamplona, Spain; 7 CIBER Fisiopatología de la Obesidad y Nutrición (CIBERobn), Instituto de Salud Carlos III, Madrid, Spain; 8 Department of Biochemistry and Genetics, University of Navarra, Pamplona, Spain; University of Barcelona, Faculty of Biology, Spain

## Abstract

**Context:**

Telomeres are biomarkers of biological aging. Shorter telomeres have been associated with increased adiposity in adults. However, this relationship remains unclear in children and adolescents.

**Objective:**

To evaluate the association between telomere length (TL) and adiposity markers in overweight/obese adolescents after an intensive program. We hypothesize that greater TL at baseline would predict a better response to a weight loss treatment.

**Design, Setting, Patients and Intervention:**

The EVASYON is a multidisciplinary treatment program for adolescents with overweight and obesity that is aimed at applying the intervention to all possibly involved areas of the individual, such as dietary habits, physical activity and cognitive and psychological profiles. Seventy-four participants (36 males, 38 females, 12–16 yr) were enrolled in the intervention program: 2 months of an energy-restricted diet and a follow-up period (6 months).

**Main Outcome:**

TL was measured by quantitative real-time polymerase chain reaction at baseline and after 2 months; meanwhile, anthropometric variables were also assessed after 6 months of follow-up.

**Results:**

TL lengthened in participants during the intensive period (+1.9±1.0, p<0.001) being greater in overweight/obese adolescents with the shortest telomeres at baseline (r = −0.962, p<0.001). Multivariable linear regression analysis showed that higher baseline TL significantly predicted a higher decrease in body weight (B = −1.53, p = 0.005; B = −2.25, p = 0.047) and in standard deviation score for body mass index (BMI-SDS) (B = −0.22, p = 0.010; B = −0.47, p = 0.005) after the intensive and extensive period treatment respectively, in boys.

**Conclusion:**

Our study shows that a weight loss intervention is accompanied by a significant increase in TL in overweight/obese adolescents. Moreover, we suggest that initial longer TL could be a potential predictor for a better weight loss response.

## Introduction

Telomeres are tandem TTAGGG repeats of DNA that, together with associated protein factors, cap the ends of chromosomes and promote chromosome stability [1]. For individuals at any age, telomere length (TL) depends on, first, the initial setting of TL in the newborn, and second, the magnitude of telomere erosion from birth onwards [2]. Telomere attrition, in turn, depends on cell replication rate, cumulative exposure to agents that produce DNA damage (such as oxidative, inflammatory, endocrine and other forms of biological stress), and activity of the telomerase enzyme [3].

The prevalence of obesity is increasing rapidly worldwide, with a high impact on children and adolescents [4]. Therefore, treating obesity in young people is critical to prevent adult obesity-related complications [5]. It is well known that obesity is characterized as a state of chronic inflammation and heightened oxidative stress [6], [7]. However, existing data on the relationship between obesity and TL in adults have yielded equivocal results; several studies reported an inverse association of TL with obesity [8]–[11], but others did not [12], [13]. Differing outcomes have also been found in pediatric population. Zannolli *et* *al*. [14] found no difference in TL between obese and normal-weight Caucasian children, whereas Al-Attas *et* *al.* [15] and Buxton *et* *al.* [16] reported significantly shorter TL in obese children compared with the nonobese ones. There is just one study regarding the relationship between adiposity and TL in adolescents that showed no association, but it demonstrated that race and sex differences in TL have already emerged during adolescence [17].

To date, a limited number of studies have explored the association between TL and adiposity indices after a lifestyle intervention, suggesting that maintaining or losing weight can lead to preservation or lengthening of the TL [10], [11], [18], [19]. Moreover, only two prospective studies considered TL as a biomarker for adiposity changes in an elderly population [9], [11].

To our knowledge, no prospective studies have assessed the relationship between TL and changes in adiposity traits in children or adolescents following a lifestyle educational program. Thus, the aim of this study was to assess the relationship between baseline TL and changes in anthropometric and obesity parameters after 2 and 6 months of a multidisciplinary intervention in overweight/obese adolescents. We hypothesize that greater TL at baseline would predict a better response to the multidisciplinary intervention, as has been previously observed in adult studies [11].

## Subjects and Methods

### Ethics Statement

Written consent to participate was requested from both parents and adolescents. The study protocols were performed in accordance with the ethical standards laid down in the 1961 Declaration of Helsinki (as revised in South Korea in 2008), following the European Economic Community (EEC) Good Clinical Practice guidelines (document 111/3976/88 of July 1990) and current Spanish law, which regulates clinical research in humans (Royal Decree 561/1993 regarding clinical trials). The study protocol was approved by the institutional review board and the Ethics Committee of each hospital that participated in this project (Madrid, Granada, Pamplona, Zaragoza, Santander) and by the Ethics Committee of the Spanish Council for Scientific Research (CSIC).

### Study Population and Intervention

The study population included 204 overweight or obese adolescents within the EVASYON program; Development, Implementation, and Evaluation of the Efficacy of a Therapeutic Program for Adolescents with Overweight and Obesity: Integral Education on Nutrition and Physical Activity (EVASYON) study (http://www.estudioevasyon.com/). EVASYON study was carried out in 5 Spanish cities (Granada, Madrid, Pamplona, Santander, and Zaragoza) and it is a lifestyle education program supported by a multidisciplinary team of nutritionists, physical education specialists, psychologists, and pediatricians. The study was implemented in two stages: an intensive, energy-restricted period for the first 2 months, and an extensive body-weight follow-up period for the last 11 months.

In the present study, we present data from the intensive treatment period corresponding to the first 2 months (an energy-restricted phase) and 6 months regarding the extensive intervention, in a subsample of 74 participants (49% males) from whom DNA samples were available. We could not do the analysis at 12 months of follow-up due to the observed drop-out rate (26%). 36 males (2.8% were overweight and 97.2% obese) and 38 females (5.3% overweight and 94.7% obese) were enrolled. The study included 12- to 16-year-old overweight or obese adolescents, according to Cole’s criteria [20], who were brought up in Spain (inclusion criteria), had no diagnosed disease associated with obesity, were not receiving pharmacologic treatment and were not diagnosed of anorexia, bulimia or other eating disorders (exclusion criteria). Cole *et* *al.* [20] developed an internationally acceptable definition of child overweight and obesity, specifying the measurement, the reference population, and the age and sex specific cut off points. They obtained the reference population by averaging across a heterogeneous mix of surveys from different countries, with widely differing prevalence rates for obesity, whereas the appropriate cut off point was defined in body mass index (BMI) units in young adulthood and extrapolated to childhood, conserving the corresponding centile in each dataset.

Based on food intake questionnaires, a personalized diet (30% of energy (E) from fat, 15% E from proteins, and 55% E from carbohydrates) [21] was prescribed while the physical activity program was instructed to each adolescent. During the intensive program period, the adolescents attended weekly group sessions where they received nutritional education, dietary advice, physical activity recommendation, as well as psychological support. During the extensive body-weight maintenance period, adolescents attended monthly in person follow-up visits with the registered dietician. The participants and their families received group sessions on different aspects such as diet, physical activity, healthy habits and weight maintenance skills, how to engage in healthy weight control behaviors and relapse prevention. The description of the complete EVASYON study has been previously published elsewhere [22], [23].

Weight and height were measured with an electronic scale (Type SECA 861; SECA, Hamburg, Germany) and with a telescopic height measuring instrument (Type SECA 225), respectively. BMI was calculated as weight (kg)/height^2^ (m^2^), then, individual BMI values were converted into SDS using age and specific cut-points according to the Spanish children and adolescent growth references [24]. Skinfolds were measured on the left side of the body to the nearest 0.1 mm with a skinfold caliper (Caliper Holtain; Holtain Ltd., Walles, UK) at triceps, biceps, subscapular, suprailiac, thigh, and calf. The waist and hip circumferences were measured with a flexible non-stretchable measuring tape (Type SECA 200). Fat mass percentage was calculated according to the Slaughter formula [25].

### Telomere Length Assessment

TL was measured in genomic DNA extracted from human peripheral blood samples, using a real-time quantitative polymerase chain reaction (RT-PCR), as described by Cawthon [26]. Telomeres were measured at two points: at baseline and after 2 months of the intensive intervention period. Concentrations of telomere repeat copy number (T) and single-copy gene (Ribosomal Protein Large PO) copy number, (S) as a reference for each sample, were obtained with this method.

PCRs were performed on white 384-well plates on an ABI-Applied Biosystems 7900 HT thermal cycler (Applied Biosystems, Austin, TX, USA). The total reaction volume was 10 µL containing 10 ng of genomic DNA, and a quantiTect Syber Green PCR kit (Qiagen, Valencia, CA, USA) was used as master mix. The final telomere primer concentrations were as follows: for telomere amplification tel1, 675 nmol/L and for tel2, 1350 nmol/L; and for the amplification of the single copy gene RPLPO: hRPLPO1, 800 nmol/L; hRPLPO2, 800 nmol/L. The primer sequences (Sigma-Aldrich, St.Louis, MO, USA) were tel1 (5′-GGTTTTTGAGGGTGAGGGTGAGGGTGAGGGTGAGGGT-3′), tel2 (5′-TCCCGACTATCCCTATCCCTATCCCTATCCCTATCCCTA-3′), hRPLPO1 (5′-CCCATTCTATCATCAACGGGTACAA -3′) and hRPLPO2 (5′-CAGCAAGTGGGAAGGTGTAATCC -3′). This method normalizes T to S by taking the ratio (T/S ratio) for each sample. The T/S ratio was calculated as follows [2^CT(telomeres)/^2^CT(single copy gene)^] = 2^−ΔCT^, since the amount of the PCR product approximately doubles in each cycle of the PCR [26].

A calibration curve of the same DNA sample of reference (64–0.25 ng in 2-fold dilutions) was included for each measurement as a standard, to control the day-to-day variations. Standard curve with linearity R^2^>0.98 was accepted. For quality control, all samples were run in triplicate and checked for concordance between triplicate values. Moreover the two DNA samples of each patient (at baseline and after 2 months of recruitment) were run in the same plate. In order to obtain a robust consistence, samples showing a high variation (more than 10%) were rerun and reanalyzed. The intra-assay coefficient of variation between triplicates was 1.6% and the inter-assay coefficient of variation between plates was 3.8%, which supports the power of this procedure.

### Statistical Analysis

The sample size calculation led to conclude that at least 22 subjects were needed. This estimate was based on the following assumptions: a two-tailed alpha error of 5%, a power of 90%, and a mean (± standard deviation) difference of 50±50 in TL after the intervention. Our study involved 74 subjects from the EVASYON study who had DNA samples at baseline and after 2 months.

We compared the anthropometric measures of the adolescents at baseline and during the weight loss treatment, stratifying the sample by sex. We calculated means and SD for each variable and assessed the statistical significance of the differences among them with repeated-measures ANOVA. We used the unpaired t-test for comparing the parameters at baseline between boys and girls.

TL was ln-transformed to achieve a better normal distribution conformation. A paired t-test was performed to evaluate the increase in TL after 2 months of the intensive intervention period, dividing the participants by sex. In addition, we also calculated the Pearson correlation coefficient (r) between baseline TL and the change in TL after the intensive treatment period after adjusting for age and sex.

We fitted multivariable linear regression modeling to analyze changes in the anthropometric variables according to the increase of 1 SD in baseline TL. Comparisons were done separately for girls and boys and were adjusted for age, baseline BMI-SDS and for each basal anthropometric variable depending on the analysis. Moreover, we also assessed differences in the adiposity traits after 2 and 6 months in males, according to the median of baseline TL. B coefficients and 95% confidence intervals (CI) were calculated using those who had the shortest telomeres at baseline as the reference group and multiple testing correction (Benjamini-Hochberg) was used to control the overall two-sided alpha level at 0.05.

Statistical analyses were performed using STATA version 12.0 (StataCorp, College Station, TX, USA). The significance level for all the analyses was set at α = 0.05 and all the tests were two-sided.

## Results

Table 1 summarizes the general features of the 74 overweight/obese adolescents (mean age 14.3±1.0 yr, 49% males) at baseline and after 2 and 6 months of the weight loss program, dividing the population by sex. Sex differences were found at baseline regarding abdominal obesity (p<0.050). Both males and females did significantly lose body weight (p<0.001) after the intervention, which was accompanied by a significant improvement in the anthropometric measurements.

**Table 1:** Anthropometric measures of the overweight/obese adolescents at baseline and during the weight loss treatment, stratifying by sex.

		BOYS				GIRLS			
	Baseline	2 months	6 months	P-value^1^	Baseline	2 months	6 months	P-value^1^	P-value^2^
**n**	36				38				
**Age**	14.3 (0.9)				14.4 (1.1)				0.512
**Weight (kg)**	85.9 (15.2)	81.2 (14.4)	78.4 (13.6)	<0.001	84.3 (14.9)	80.6 (13.7)	81.1 (13.5)	<0.001	0.650
**BMI-SDS**	5.0 (1.6)	4.1 (1.7)	3.2 (1.6)	<0.001	4.6 (2.0)	3.9 (1.7)	3.9 (1.9)	<0.001	0.363
**Body fat (%)**	47.3 (8.5)	42.8 (8.1)	39.2 (8.4)	<0.001	44.1 (6.4)	40.1 (6.7)	40.3 (5.5)	<0.001	0.082
**Waist circumference (cm)**	101.9 (9.8)	98.5 (10.6)	92.0 (10.1)	<0.001	108.4 (12.0)	104.3 (10.6)	102.6 (12.4)	<0.001	0.014
**Hip circumference (cm)**	101.6 (22.8)	99.0 (20.8)	95.1 (19.6)	<0.001	85.9 (27.4)	81.5 (23.9)	81.8 (22.9)	0.133	0.011
**Waist to hip ratio**	1.08 (0.38)	1.06 (0.36)	1.03 (0.35)	0.087	1.39 (0.46)	1.39 (0.42)	1.36 (0.43)	0.695	0.002
**Waist to height ratio**	0.61 (0.05)	0.59 (0.06)	0.54 (0.05)	<0.001	0.67 (0.07)	0.64 (0.06)	0.63 (0.08)	<0.001	<0.001
**Σ 6 skinfolds (mm)**	182.9 (24.9)	166.5 (28.8)	150.2 (37.0)	<0.001	194.6 (27.6)	179.3 (28.3)	176.9 (31.5)	<0.001	0.113

The table shows means (SD). BMI-SDS: Standard Deviation Score for BMI.

1 : p value in three different time points analyzed by repeated-measures ANOVA in subjects distributed by sex.

2 : p value for the comparison at baseline between boys and girls.

**Figure 1:**
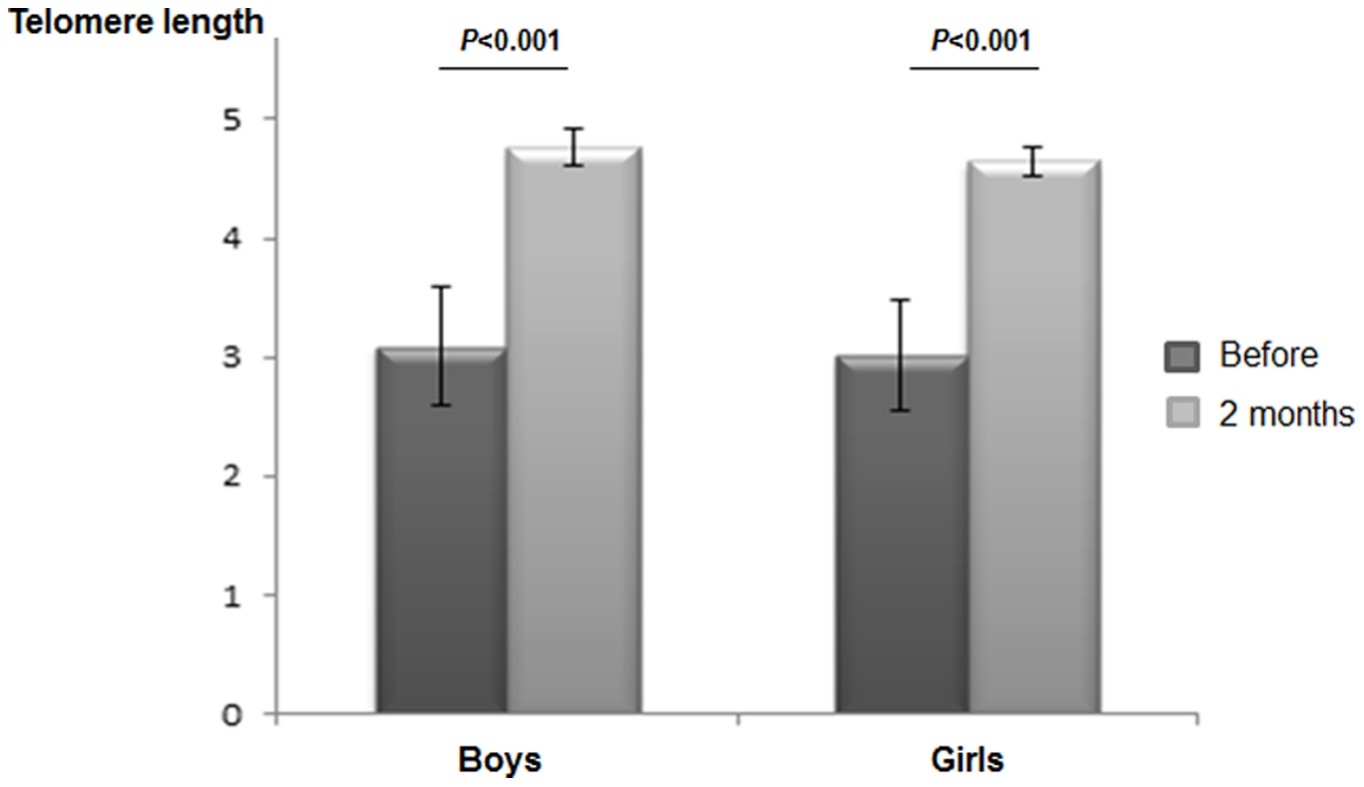
Telomere length distribution in boys and girls before and after the intensive lifestyle intervention. Significant differences were found after 2 months *vs.* before the multidisciplinary program.

Average TL was ascertained in 74 subjects using RT-PCR. The analyses were carried out after ln-transformed of all T/S ratios. Interestingly, TL significantly increased after the intensive lifestyle intervention in 88% of the adolescents (+1.93 [1.69−2.18], p<0.001) (data not shown). In this sense, mean differences values (95% CI) were +1.66 (1.27−2.06) for boys and +1.64 (1.22−2.06) among girls (Figure 1). No differences in TL were found at baseline or during the 2 months of intensive intervention period in subjects according to sex.

**Figure 2:**
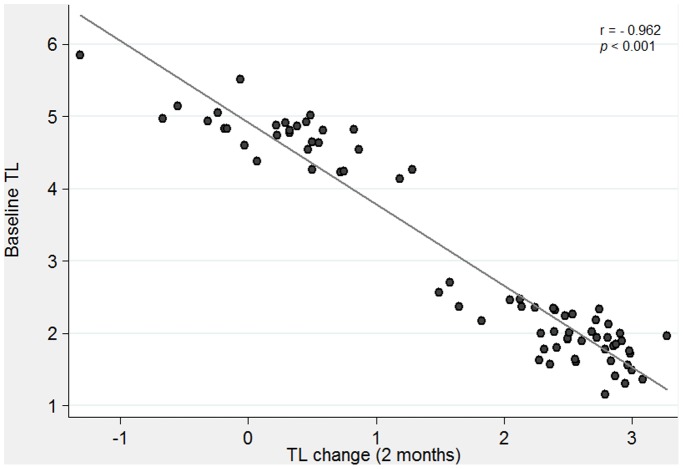
Telomere length change after the intensive intervention as a function of telomere length at baseline. The scatter plot shows the entire adolescent’s sample (n = 74), each dot representing one individual. Relative telomere length is adjusted for age and sex.

Furthermore, the individual change in TL was inversely correlated with initial TL at a highly significant level after controlling for age and sex (r = −0.962, p<0.001; Figure 2), indicating that the lengthening rate was most pronounced in individuals with shorter telomeres at baseline. Due to this strong correlation, baseline TL was considered as a potential predictor factor for changes in adiposity in obese adolescents. Surprisingly, we did not observe a significant association between TL and participant’s age (r = −0.122, p = 0.301; data not shown).

We fitted a multiple regression model to predict the changes in anthropometric and adiposity traits at follow-up according to sex (Table 2). In boys, during 2 months of the intensive treatment phase, higher baseline TL significantly predicted a greater reduction in body weight (B = −1.53, 95% CI: −5.57 to −0.49) and BMI-SDS (B = −0.22, 95% CI: −0.38 to −0.05) after adjusting for age and the respective variable at baseline. Moreover, a discernible trend was observed in waist circumference (B = −1.47, 95% CI:−3.04 to 0.09), hip circumference (B = −1.83, 95% CI:−3.78 to 0.12), and waist to height ratio (B = −0.01, 95% CI:−0.02 to 0.001). However, no differences were observed in girls. Interestingly, males with higher initial TL also presented a greater decrease in body weight (B = −2.25, 95% CI:−4.48 to −0.03) and BMI-SDS (B = −0.47, 95% CI:−0.79 to −0.15) after 6 months of the multidisciplinary intervention.

**Table 2:** Changes in anthropometric variables after 2 and 6 months of the weight loss program, according to the increase of 1

	Baseline TL							
	Boys (n = 36)				Girls (n = 38)			
	2 months		6 months		2 months		6 months	
	B (95% CI)	P-value	B (95% CI)	P-value	B (95% CI)	P-value	B (95% CI)	P-value
**Δ Body Weight (kg)**								
Unadjusted	−1.35	0.017	−2.08	0.086	0.44	0.487	0.27	0.767
	(−2.45 to −0.25)		(−4.48 to 0.31)		(−0.84 to 1.73)		(−1.59 to 2.13)	
Adjusted^1^	−1.53	0.005*	−2.25	0.047	0.84	0.149	0.71	0.406
	(−5.57 to −0.49)		(−4.48 to −0.03)		(−0.32 to 2.01)		−(1.01 to 2.43)	
**Δ BMI-SDS**								
Unadjusted	−0.20	0.014	−0.46	0.006	0.10	0.484	0.03	0.780
	(−0.35 to −0.04)		(−0.78 to −0.14)		(−0.18 to 0.38)		(−0.22 to 0.29)	
Adjusted^1^	−0.22	0.010*	−0.47	0.005*	0.20	0.102	0.09	0.443
	(−0.38 to −0.05)		(−0.79 to −0.15)		(−0.04 to 0.43)		(−0.15 to 0.34)	
**Δ Body fat (%)**								
Unadjusted	0.20	0.750	−0.45	0.686	0.99	0.246	0.98	0.252
	(−1.08 to 1.49)		(−2.68 to 1.79)		(−0.71 to 2.69)		(−0.72 to 2.68)	
Adjusted^1^	0.14	0.826	−0.75	0.482	1.15	0.152	0.95	0.146
	(−1.13 to 1.41)		(−2.91 to 1.41)		(−0.45 to 2.75)		(−0.35 to 2.25)	
**Δ Waist circumference (cm)**								
Unadjusted	−1.98	0.037	−1.85	0.257	0.74	0.577	2.04	0.191
	(−3.48 to −0.12)		(−5.12 to 1.41)		(−1.94 to 3.42)		(−1.06 to 5.14)	
Adjusted^1^	−1.47	0.065	−1.56	0.304	1.38	0.221	2.73	0.078
	(−3.04 to 0.09)		(−4.59 to 1.48)		(−0.87 to 3.62)		(−0.32 to 5.78)	
**Δ Hip** **circumference (cm)**								
Unadjusted	−0.72	0.505	0.50	0.725	3.86	0.125	4.70	0.067
	(−2.88 to 1.45)		(−2.38 to 3.38)		(−1.12 to 8.85)		(−0.35 to 9.74)	
Adjusted^1^	−1.83	0.065	−0.92	0.409	2.52	0.260	1.58	0.493
	(−3.78 to 0.12)		(−3.17 to 1.33)		(−1.96 to 6.99)		(−3.05 to 6.21)	
**Δ Waist to hip**								
Unadjusted	−0.01	0.461	−0.04	0.021	−0.05	0.103	−0.09	0.051
	(−0.05 to 0.02)		(−0.09 to −0.01)		(−0.11 to 0.01)		(−0.18 to 0.01)	
Adjusted^1^	0.01	0.914	−0.03	0.084	−0.04	0.209	−0.05	0.309
	(−0.03 to 0.04)		(−0.07 to 0.01)		(−0.09 to 0.02)		(−0.14 to 0.04)	
**Δ Waist to height**								
Unadjusted	−0.01	0.046	−0.01	0.245	0.005	0.499	0.01	0.220
	(−0.02 to −0.001)		(−0.03 to 0.01)		(−0.01 to 0.02)		(−0.01 to 0.03)	
Adjusted^1^	−0.01	0.078	−0.01	0.251	0.01	0.117	0.01	0.119
	(−0.02 to 0.001)		(−0.03 to 0.01)		(−0.01 to 0.02)		(−0.01 to 0.03)	
**Δ Σ 6 skinfolds (mm)**								
Unadjusted	−2.92	0.306	−4.08	0.347	4.04	0.300	7.37	0.220
	(−8.63 to 2.79)		(−12.77 to 4.61)		(−3.75 to 11.83)		(−0.71 to 15.46)	
Adjusted^1^	−2.18	0.440	−3.79	0.411	4.41	0.237	7.48	0.059
	(−7.87 to 3.51)		(−13.06 to 5.48)		(−3.04 to 11.87)		(−0.30 to 15.25)	

TL: telomere length, BMI-SDS: Standard Deviation Score for BMI, SD: Standard Deviation.

1 Adjusted for age, basal BMI-SDS and the respective variable at baseline.

*P-value <0.05 after correcting for Benjamini–Hochberg multiple comparisons.

In addition, when we dichotomized the male sample at the median of baseline TL, those who presented longer telomeres displayed a greater reduction in body weight (p = 0.001), BMI-SDS (p = 0.004) and hip circumference (p = 0.012), and they showed a similar trend in waist circumference (p = 0.067), and waist to height ratio (p = 0.061) after the intensive lifestyle program (Table 3). Similarly, after 6 months of the intervention, the longer the initial TL, the higher the decrease in body weight (p = 0.014) and BMI-SDS (p = 0.002).

**Table 3:** Multivariable-adjusted differences (95% confidence intervals) in the change of the anthropometric measures, by the median of baseline telomere length in boys.

	Baseline TL		
	<2.34	≥2.34	P-value
	(n = 18)	(n = 18)	
***Change in obesity traits***			
**Body weight (kg)**			
2 months	−2.85 (−4.21 to −1.49)	−6.54 (−7.89 to −5.18)	0.001*
6 months	−3.99 (−7.04 to −0.95)	−9.67 (−12.62 to −6.71)	0.014*
**BMI-SDS**			
2 months	−0.68 (−0.89 to −0.46)	−1.16 (−1.38 to −0.95)	0.004*
6 months	−1.06 (−1.51 to −0.62)	−2.12 (−2.55 to −1.69)	0.002*
**Waist circumference (cm)**			
2 months	−1.97 (−4.08 to 0.23)	−4.89 (−7.05 to −2.74)	0.067
6 months	−8.79 (−12.99 to 4.59)	−11.23 (−15.43 to −7.03)	0.429
**Hip circumference (cm)**			
2 months	−0.20 (−2.70 to 2.29)	−5.10 (−7.60 to −2.60)	0.012*
6 months	−5.56 (−8.57 to −2.54)	−7.51 (−10.53 to −4.50)	0.388
**Waist to hip ratio**			
2 months	−0.03 (−0.08 to 0.02)	−0.01 (−0.05 to 0.04)	0.450
6 months	−0.03 (−0.08 to 0.02)	−0.07(−0.12 to −0.02)	0.262
**Waist to height ratio**			
2 months	−0.01 (−0.03 to −0.001)	−0.03 (−0.05 to −0.02)	0.061
6 months	−0.06 (−0.09 to −0.04)	−0.08 (−0.10 to −0.06)	0.326
**Σ 6 skinfolds (mm)**			
2 months	−14.07 (−21.90 to−6.25)	−18.62 (−26.45 to −10.80)	0.429
6 months	−28.85 (−41.59 to −16.10)	−36.60 (−49.35 to −23.86)	0.408

Data is presented as B (95%CI). TL: telomere length, BMI-SDS: Standard Deviation Score for BMI.

Adjusted for age, basal BMI-SDS and the respective variable at baseline.

*P-value <0.05 after correcting for Benjamini–Hochberg multiple comparisons.

## Discussion

In this study encompassing 74 overweight/obese adolescents, a significant increase in TL after 2 months of a multidisciplinary program was found. Interestingly, we observed a significant association between baseline TL and changes in adiposity traits in boys which remained significant after 6 months of follow-up. To our knowledge, this study assessed for the first time the relationship between TL and changes in obesity traits in an obese adolescent population.

We did not observe differences in TL according to age or sex, in agreement with studies in children and adolescent populations [15], [16]. The narrow age range of our adolescent population (12–16 years) and the relatively small sample size did not favor this age-related phenomenon.

Interestingly, we showed that an integral intervention for weight loss may contribute to the prevention of telomere shortening. In adults, a 4-month supplementation with omega-3 did increase leukocyte TL [27]. In other cell types an increase in TL was observed after 1 or 3 months dietary intervention in adult subjects [18], [28]. Two potential mechanisms could explain this telomere lengthening: a reduction in biochemical stress or a replenishment of younger cells with longer telomeres into circulation [29]. However, our findings need to be further explored in other populations to better understand this biological mechanism.

Besides, subjects who had the shortest telomeres at baseline presented the greatest increase in TL that is in concordance with other adult studies [30]–[32]. This finding might be due to TL maintenance machinery which is focused on protecting the shortest telomeres [33]–[35]. It could be speculated that obese adolescents with longer telomeres at baseline had a lower rate of telomere lengthening, since the change in TL mainly depends on the initial TL, achieving a greater weight loss.

Few epidemiologic studies have investigated the association of TL and obesity traits in young population [14]–[17]. In adolescents a cross-sectional study did not observe association between TL and adiposity indices [17]. Obese children (French and Arab) showed shorter telomeres than their nonobese counterparts [15], [16]. In Italian children no difference in TL between obese and nonobese children was found [14]. However, our findings give valuable information in a longitudinal way.

This study proposed TL as a biomarker for adiposity changes, as we showed that the longer the initial telomere, the greater the decrease in obesity parameters. In this regard, Njajou *et* *al.* [9] observed TL to be significantly associated with percentage of change in BMI and in body fat during a 7-year follow-up in the elderly. In the frame of the PREDIMED study, we recently reported that higher initial TL could predict a greater decrease in obesity anthropometric variables in an elderly population [11].

Furthermore, significant associations between TL and changes in adiposity indices were observed only in boys. The sex effect elicited in this study confirms the findings of Al-Attas *et* *al.* who observed a relationship between obesity and TL in boys aged 5–12 years [15]. Nevertheless, the possible explanation for our sex difference might be due to biological sex differences that may modulate this association. In fact, our participants were at a high growing rate where hormones, particularly in girls, could play an important role.

Some limitations should be acknowledged. There is a debate on whether observed telomere lengthening is real or an artifact caused by measurement errors. Thus, Steenstrup *et* *al.* [36] showed that the effect of measurement error can be reduced in longitudinal studies by presenting meticulous attention to potential measurement problems. Therefore, we have carefully controlled the experimental conditions to avoid potential errors: genomic DNA was processed following a standardized protocol to preserve its stability; and also the two DNA samples per patient (at baseline and after 2 months) were run in the same plate. Nevertheless, it should be recognised that our study consisted in a short intervention period leading to the possibility of individuals to be misclassified as TL gainers [36]. The small sample size and the progressive pubertal stages among participants are also weaknesses of this study. However, the fact that important statistical differences were found suggests that potential type-II errors were overcome. On the other hand, strengths of our study include: 1) the design allows reproducing real-time conditions with home-prepared foods in free-living individuals, as in usual clinical practice; 2) measurements in young subjects are not confounded by chronic obesity-related disorders.

In conclusion, our results suggest that a multidisciplinary intervention in obese adolescents did achieve not only weight loss, but an increase in TL. Moreover, we show that initial longer telomeres could predict a better weight loss response to a multidisciplinary intervention program in overweight/obese male adolescents. However, further larger longitudinal studies are warranted to confirm these results and better understand this complicated association, especially at a young age in order to prevent adult obesity-related complications.
